# Implication of Potential Differential Roles of the Two Phosphoglucomutase Isoforms in the Protozoan Parasite *Cryptosporidium parvum*

**DOI:** 10.3390/pathogens11010021

**Published:** 2021-12-24

**Authors:** Jiawen Nie, Jigang Yin, Dongqiang Wang, Chenchen Wang, Guan Zhu

**Affiliations:** Key Laboratory of Zoonosis Research of the Ministry of Education, the Institute of Zoonosis, and the College of Veterinary Medicine, Jilin University, Changchun 130062, China; niejw18@mails.jlu.edu.cn (J.N.); yinjg@jlu.edu.cn (J.Y.); wdq19@mails.jlu.edu.cn (D.W.); wcc18@mails.jlu.edu.cn (C.W.)

**Keywords:** *Cryptosporidium*, apicomplexan, phosphoglucomutase 1 (PGM1), signal peptide, enzyme kinetics, glycolysis, glucogenesis, glycolytic enzyme

## Abstract

Phosphoglucomutase 1 (PGM1) catalyzes the conversion between glucose-1-phosphate and glucose-6-phosphate in the glycolysis/glucogenesis pathway. PGM1s are typically cytosolic enzymes in organisms lacking chloroplasts. However, the protozoan *Cryptosporidium* parasites possess two tandemly duplicated *PGM1* genes evolved by a gene duplication after their split from other apicomplexans. Moreover, the downstream PGM1 isoform contains an N-terminal signal peptide, predicting a non-cytosolic location. Here we expressed recombinant proteins of the two PGM1 isoforms from the zoonotic *Cryptosporidium parvum*, namely CpPGM1A and CpPGM1B, and confirmed their enzyme activity. Both isoforms followed Michaelis–Menten kinetics towards glucose-1-phosphate (*K*_m_ = 0.17 and 0.13 mM, *V*_max_ = 7.30 and 2.76 μmol/min/mg, respectively). *CpPGM1A* and *CpPGM1B* genes were expressed in oocysts, sporozoites and intracellular parasites at a similar pattern of expression, however *CpPGM1A* was expressed at much higher levels than *CpPGM1B*. Immunofluorescence assay showed that CpPGM1A was present in the cytosol of sporozoites, however this was enriched towards the plasma membranes in the intracellular parasites; whereas CpPGM1B was mainly present under sporozoite pellicle, although relocated to the parasitophorous vacuole membrane in the intracellular development. These observations indicated that CpPGM1A played a house-keeping function, while CpPGM1B played a different biological role that remains to be defined by future investigations.

## 1. Introduction

*Cryptosporidium* is a genus of protozoan parasites of humans and other vertebrates. Humans are mainly infected by the zoonotic *C. parvum* and anthroponotic *C. hominis*, while animals including farm animals may be infected by different *Cryptosporidium* species with varied host specificities [1,2,3,4]. The main symptom of cryptosporidiosis with clinical significance in humans and other mammals is watery diarrhea, which may range from mild to severe or fatal in hosts with weakened immunity. One of the top five pathogens causing moderate to severe diarrhea, it is a significant cause of mortality and morbidity in infants and children in resource-restricted countries or regions [2,5]. However, there is still a lack of fully effective treatments for cryptosporidiosis in humans and animals [2,6,7].

As a member under the Phylum Apicomplexa, *Cryptosporidium* is evolutionarily branched early at the base of the Phylum, making it highly divergent from other apicomplexans such as *Toxoplasma*, *Eimeria* and *Plasmodium* species at cellular and molecular levels [8,9,10]. For example, in contrast to the coccidia and hematozoa, intestinal *Cryptosporidium* species (e.g., *C. parvum* and *C. hominis*) lacks Krebs cycle and cytochrome-based respiratory chain; while gastric species (e.g., *C. muris* and *C. andersoni*) possess Krebs cycle, yet lacks respiratory chain [8,11,12]. Therefore, *Cryptosporidium* species maintain an “anaerobic” parasitic lifestyle, solely or mainly relying on glycolysis to produce ATP. *Cryptosporidium* produces amylopectin to store energy, uses amylopectin and hexoses (e.g., glucose) to start and releases three organic products (i.e., lactate, ethanol and acetate) to end the glycolytic pathway [8,11].

**Figure 1:**
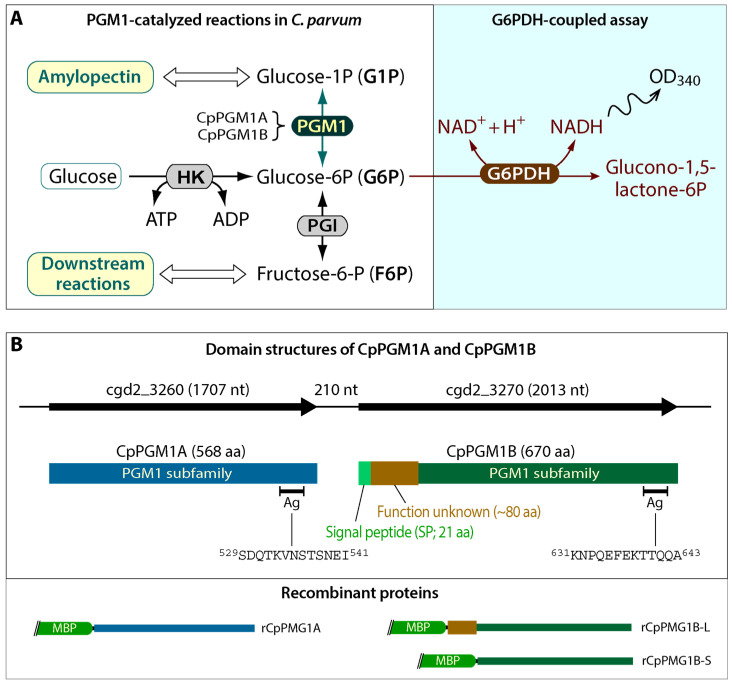
Function of phosphoglucomutase 1 (PGM1) and domain organization of CpPGM1A and CpPGM1B. (**A**) Left panel: PGM1-catalyzed reaction in the glycolysis and glucogenesis of *Cryptosporidium parvum*. Right panel: Glucose-6P dehydrogenase (G6PDH) was not part of the pathway in the parasite, however this was used to convert glucose-6P (G6P) to glucono-1,5-lactone-6P using NAD^+^ as an electron receiver in the G6PDH-coupled assay for detecting PGM1 activity. The production of NADH was detected spectrophotometrically at 340 nm. HK, hexokinase. (**B**) Upper panel: Illustration of the organization of *CpPGM1A* (cgd2_3260) and *CpPGM1B* (cgd2_3270) genes in the parasite chromosome 2 and the domain structures of their products (CpPGM1A and CpPGM1B proteins). The antigen (Ag) sites for producing polyclonal antibodies and their amino acid sequences are also labeled. Lower panel: The two parasite PGM1 isoforms were expressed as maltose-binding protein (MBP)-fusion proteins marked as rCpPGM1A and rCpPGM1B, respectively. More detailed alignments and annotations of functionally important amino acid residues are included in Figures S1 and S2.

In glycolysis/glucogenesis, phosphoglucomutase (PGM) [EC: 5.4.2.2] is a key enzyme at the intersection between the synthesis and degradation of starch, or amylopectin in the case of *Cryptosporidium*, by catalyzing the conversion between glucose-1-phosphate (G1P) and glucose-6-phosphate (G6P) (Figure 1A). *Cryptosporidium* parasites possess two tandemly duplicated PGM-encoding genes that are highly homologous at both nucleotide and protein levels (Figures S1 and S2). The presence of two PGM genes seems to be unusual for *Cryptosporidium* parasites that possess the smallest genomes and are featured by highly streamlined metabolism with little gene redundancy. In the zoonotic *C. parvum*, the two PGM enzymes (CpPGM1A and CpPGM1B) contain a classic PGM1 subfamily domain, however CpPGM1B possesses an N-terminal signal peptide (SP) followed by a “linker” sequence with unknown function (Figure 1B). The possession of SP is also uncommon for a glycolytic enzyme supposed to catalyze a reaction in the cytosol. Therefore, an intriguing question to ask is why *Cryptosporidium* parasites possess two PGMs with one of them containing an SP.

In the present study, we expressed recombinant CpPGM1A and CpPGM1B proteins and biochemically confirmed that both enzymes were enzymatically active. We also raised polyclonal antibodies for immunofluorescence labeling and observed that CpPGM1A was mainly cytosolic, while CpPGM1B was associated with membranes, in sporozoites and intracellular stages of the parasite. These observations suggest that CpPGM1A and CpPGM1B might play differential biological roles in the parasite.

## 2. Results and Discussion

### 2.1. Cryptosporidium Possessed Two Tandemly Duplicated PGM1-Subfamily Genes Predicted to Encode a Cytosolic and a Non-Cytosolic Protein

By datamining CryptoDB (https://www.cryptodb.org (accessed on 23 December 2021)), two tandemly duplicated genes encoding PGM proteins were observed in all available *Cryptosporidium* genomes. In *C. parvum*, the two PGM isoforms were encoded at the loci cgd2_3260 and cgd2_3270 and their predicted ORFs (1707 and 2013 bp, respectively) were separated by a 210-base intergenic space (Figure 1B). Based on NCBI’s conserved domain database (CDD) (https://www.ncbi.nlm.nih.gov/cdd) (accessed on 23 December 2021) and EMBL-EBI’s InterPro database (https://www.ebi.ac.uk/interpro) (accessed on 23 December 2021), the two PGM isoforms belonged to the PGM1 subfamily. As some PGM subfamilies were named by having specified numbers applied to the end of their title (e.g., PGM1 refers to enzymes catalyzing the bidirectional interconversion of G1P and G6P via a glucose 1,6-diphosphate intermediate, while PGM2 refers to phosphopentomutase catalyzing the conversion of ribose-1-phosphate or deoxyribose-1-phosphate to the corresponding 5-phosphopentoses), we named the two *C. parvum* PGM isoforms as CpPGM1A (cgd2_3260) and CpPGM1B (cgd2_3270) to clarify that they were PGM1 subfamily enzymes. Based a common practice in the field, *CpPGM1A* and *CpPGM1B* in italics were used for gene names, and CpPGM1A and CpPGM1B in non-italics refer to gene products (e.g., mRNA or proteins).

Based on domain analysis, CpPGM1A contained a classic PGM1 domain and was predicted to be cytosolic. However, CpPGM1B possessed not only a PGM1 domain, but also an N-terminal SP followed by a linker sequence (~80 aa) with no apparent homologs in other proteins (Figure 1B). The presence of an N-terminal SP in CpPGM1B was uncommon for a glycolytic enzyme, and would result in the translocation of the enzyme to the lumen of endoplasmic reticulum (ER) during the translation of protein from mRNA, making it function in a non-cytosolic location(s).

**Figure 2:**
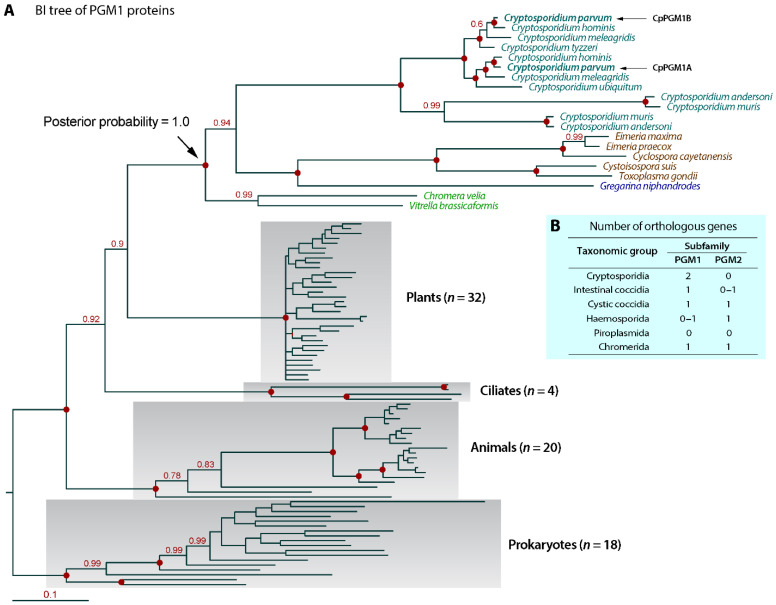
Phylogenetic relationship of CpPGM1A and CpPGM1B within apicomplexan orthologs and the numbers of apicomplexan PGM1 and PGM2 orthologs. (**A**) Bayesian inference (BI)-based phylogenetic tree inferred from PGM1 subfamily proteins (total 94 taxa) including those from apicomplexans, chromerids, ciliates, plants, animals and prokaryotes (log*L* = −25,191.19). Numbers at the major nodes are posterior probabilities. Bar indicates amino acid substitution rate. (**B**) Number of orthologous genes in the PGM1 and PGM2 subfamilies from the major taxonomic groups of apicomplexans and chromerids based on datamining the available genome sequences. *Cryptosporidium* parasites differ from other apicomplexans by possessing two duplicated PGM1 subfamily genes but lacking a PGM2 ortholog.

The PGM1 domains were highly conserved between CpPGM1A and CpPGM1B with only 52 mismatched residues out of the total 567 amino acids; or in other words, 90.83% of the amino acids were identical between CpPGM1A and CpPGM1B (Figure S1). The CpPGM1A and CpPGM1B also contained all conserved residues at the active sites, including metal and substrate binding sites (Figure S1). The *CpPGM1A* and *CpPGM1B* genes were also highly conserved at nucleotide level, sharing 91.7% identity (Figure S2). The *CpPGM1A* and *CpPGM1B* orthologs were present in synteny and all available *Cryptosporidium* genomes, including intestinal (e.g., *C. parvum*, *C. hominis* and *C. meleagridis*) and gastric (i.e., *C. muris* and *C. andersoni*) species. In a Bayesian inference-based phylogenetic analysis with strong statistical support at the major nodes by posterior probabilities, *Cryptosporidium* PGM1A and PGM1B orthologs formed a single clade separated from those of other apicomplexans (Figure 2A). Tandemly duplicated *PGM1* genes were not observed in other groups of apicomplexans that possessed either a single *PGM1* ortholog (i.e., intestinal/cystic coccidia and some haemosporids) or none at all (i.e., piroplasmids and most haemosporids) (Figure 2B). Noticeably, the genes encoding PGM2 subfamily enzymes were absent in cryptosporidia, yet were present in single copies in some other apicomplexans with the exception of piroplasmids that contained neither *PGM1* nor *PGM2* genes (Figure 2B). Collectively, we may conclude that the presence of tandemly duplicated *PGM1A* and *PGM1B* genes is unique to *Cryptosporidium* and the two genes evolved from a gene duplication event after the split of the *Cryptosporidium* genus from other apicomplexans.

### 2.2. Both CpPGM1A and CpPGM1B Were Enzymatically Active

**Figure 3:**
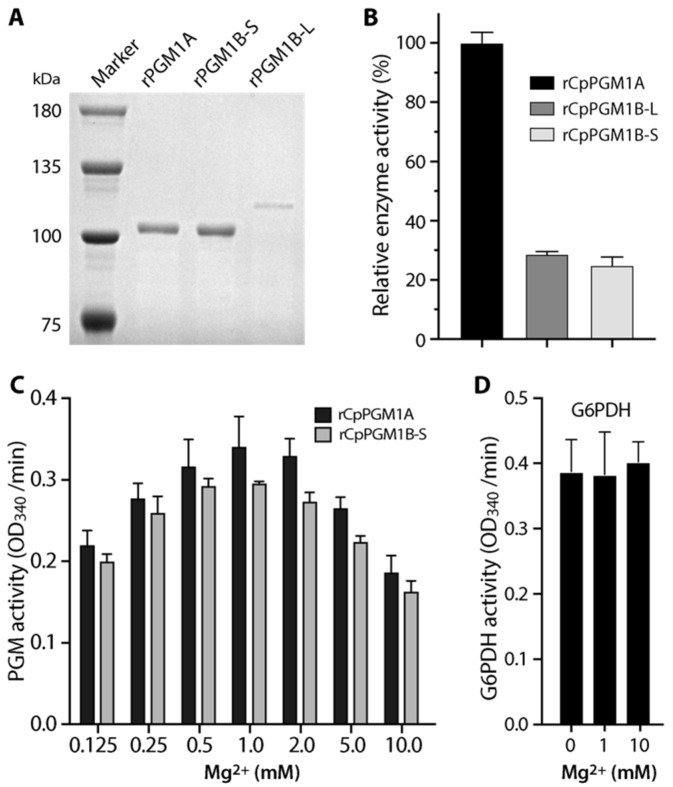
Enzymatic activity of recombinant CpPGM1A and CpPGM1B proteins. (**A**) SDS-PAGE (6% gel) analysis of purified recombinant proteins, including rCpPGM1A (product of the whole open reading frame (ORF) as MBP-fusion protein), rCpPGM1B-S (short form containing PGM1 domain) and rCpPGM1B-L (long form containing the linker sequence with unknown function and the PGM1 domain). Also see Figure 1B for illustration of the constructs of the recombinant CpPGM1 isoforms. The gel was stained with Coomassie brilliant blue. (**B**) Relative enzyme activity of the three forms of recombinant CpPGM1 isoforms, showing that both long and short forms of CpPGM1B proteins are enzymatically active, however their specific activities were much lower than that of CpPGM1A. (**C**) Relative activities of rCpPGM1A and rCpPGM1B-S in reactions containing various concentrations of Mg^2+^. (**D**) Assay showing that Mg^2+^ had no effect on the activity of G6PDH used in the G6PDH-coupled assay. All assays were performed at least three times independently. Bars represent standard errors of the mean (SEMs) derived from at least three replicated reactions.

We evaluated whether CpPGM1A and CpPGM1B were enzymatically active using MBP-fusion proteins, in which CpPGM1A was expressed in whole protein, while CpPGM1B was expressed in two forms: a long form containing linker and PGM1 domain with exclusion of SP and a short one containing only the PGM1 domain (see illustration in Figure 1B). All three recombinant proteins were successfully expressed and purified into high purity (Figure 3A). However, the expression of the long form of CpPGM1B (CpPGM1B-L) was much less efficient than the short form CpPGM1B-S. The reason was unclear, however the hydrophobicity of the 80 aa region were unlikely to be the reason based on the hydrophobic profile analysis.

Using a G6PDH-coupled assay, all three recombinant proteins (i.e., rCpPGM1A, rCpPGM1B-L and rCpPGM1B-S) displayed activity in converting G1P to G6P (Figure 3B). The relative enzymatic activities of rCpPGM1A, rCpPGM1B-L and rCpPGM1B-S were 100%, 28.6% and 24.9%, respectively. After confirming that both rCpPGM1B-L and rCpPGM1B-S were enzymatically active, the short form that was expressed more efficiently was used in subsequent experiments.

**Figure 4:**
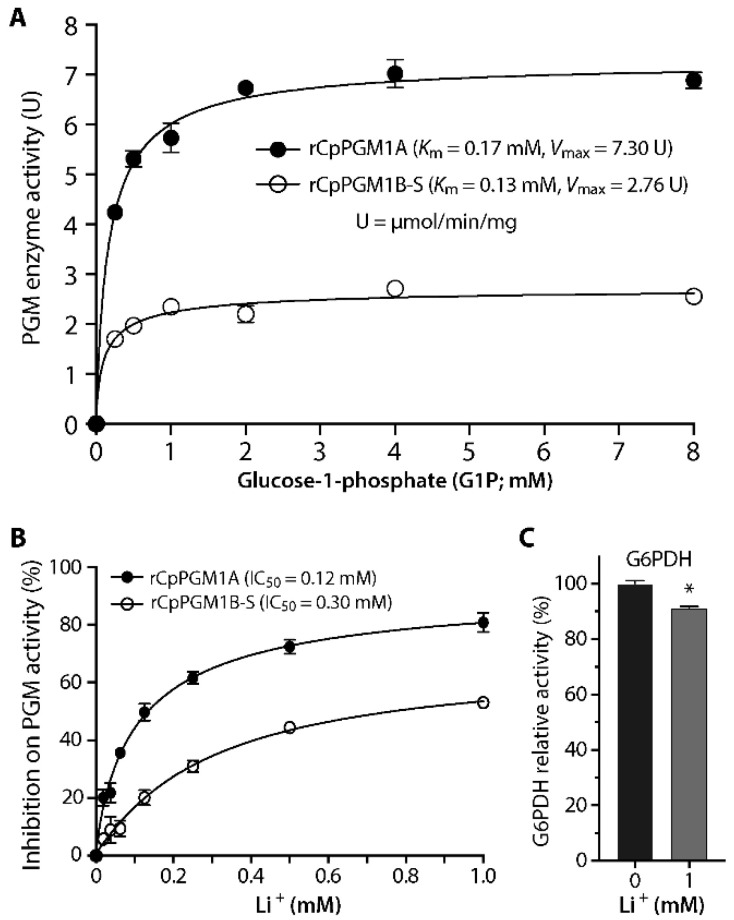
Enzyme kinetics of rCpPGM1A and rCpPGM1B-S. (**A**) Both rCpPGM1A and rCpPGM1B-S followed Michaelis–Menten kinetics with lower micromolar affinity towards substrate glucose-1P (*K*_m_ = 0.17 and 0.13 mM, respectively), but varied in specific activities (*V*_max_ = 7.30 and 2.76 μmol/min/mg, respectively). (**B**) Inhibition of Li^+^ on rCpPGM1A and rCpPGM1B-S enzyme activities (IC_50_ = 0.12 and 0.30 mM, respectively). (**C**) Assay showing Li^+^ had a small effect on the activity of G6PDH used in the G6PDH-coupled assay (i.e., 8.8% inhibition at 1 mM). All assays were performed at least three times independently. Bars represent standard errors of the mean (SEMs) derived from at least three replicated reactions. * *p* < 0.01 by Student *t*-test.

Next, we confirmed that the optimal concentrations of Mg^2+^ for both rCpPGM1A and rCpPGM1B-S were between 0.5 and 2.0 mM (Figure 3C), by which 1.0 mM of MgCl_2_ was used in subsequent experiments. We also confirmed that G6PDH coupled in the assay for producing NADH was not Mg^2+^-dependent and insensitive to the variation in Mg^2+^ concentrations (Figure 3D). We also observed that rCpPGM1A and rCpPGM1B-S followed Michaelis–Menten kinetics and determined the kinetic parameters towards substrate G1P (i.e., *K*_m_ = 0.17 and 0.13 mM, *V*_max_ = 7.30 and 2.76 μmol/min/mg, respectively) (Figure 4A). Among these parameters, the *K*_m_ values were comparable to those of PGMs from *T. gondii* PGM1 (0.19 mM) [13], *Saccharomyces cerevisiae* (0.112 to 2.0 mM) [14,15], and humans (0.081 to 0.088 mM) [16,17]. However, the specific activities were much lower than those from *T. gondii* and human PGM1 (i.e., 338 and 1263 μmol/min/mg, respectively).

Earlier studies showed that mammalian and yeast PGM1 was sensitive to Li^+^ by competing with Mg^2+^ for the metal binding site [18,19]. Here we also observed inhibition of rCpPGM1A and rCpPGM1B-S activity by Li^+^ in a dose-dependent manner (IC_50_ = 0.12 and 0.30 mM, respectively) (Figure 4B). The inhibition was specific on the parasite enzymes, as Li^+^ only showed a small effect on the activity of G6PDH in the assay, i.e., 8.8% inhibition of enzyme activity at 1 mM (Figure 4C).

### 2.3. CpPGM1A and CpPGM1B Genes Were Differentially Expressed

**Figure 5:**
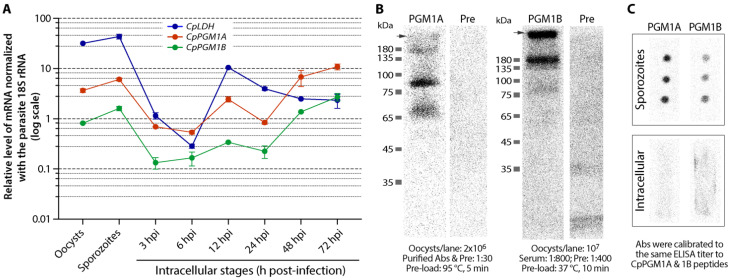
Detection of transcripts of *CpPGM1A* and *CpPGM1B* genes and their protein products. (**A**) Relative levels of *CpPGM1A* and *CpPGM1B* transcripts in various developmental stages of *C. parvum* as determined by qRT-PCR. *CpLDH* transcript was also detected in the assay as a reference and for quality control. Relative levels of transcripts were calculated based on ∆∆C_T_ values using the parasite 18S rRNA for normalization. (**B**) Western blot detection of native CpPGM1A and CpPGM1B proteins in crude extracts from the oocysts of *C. parvum*. Arrows indicate the entry points of the separating gel. The number of oocysts corresponding to the sample amount in each lane, and conditions for each specimen, were marked at the bottom of the blots. (**C**) Dot blot analysis of CpPGM1A and CpPGM1B proteins in crude extracts from sporozoites (10^5^ sporozoites/dot; upper panel) and infected host cells at 12 h post-infection (14 μg/dot; lower panel). In this assay, the concentrations of the two primary antibodies were calibrated to the same titer against their peptide antigens by ELISA.

A qRT-PCR detection of transcripts showed that *CpPGM1A* and *CpPGM1B* genes were expressed in all life cycle stages obtainable in vitro (Figure 5A). Both genes displayed a similar pattern of expression during the parasite life cycle, showing higher levels of transcripts in oocysts, sporozoites and later stages of intracellular development (i.e., 48 and 72 hpi), yet lower levels in earlier intracellular stages (i.e., 3, 6, 12 and 24 hpi). The higher level of expression might be correlated with the utilization of amylopectin as an energy source for environmental stages of the parasite (i.e., oocysts and sporozoites) or the synthesis/accumulation of amylopectin as energy storage during the formation of oocysts in the later gametogenesis and oocyst formation stages. Noticeably, the levels of transcript of *CpPGM1A* were consistently higher than those of *CpPGM1B* (i.e., between 3.23- and 7.18-fold; mean = 4.57-fold) (Figure 5A). As a reference and for quality control included in the assay, the transcript of the *CpLDH* gene showed a pattern differing from those of the *CpPGM1* genes, however followed a previously reported pattern [20,21], confirming the reliability of the qRT-PCR assay.

In the western blot analysis using crude protein extracts from oocysts, affinity purified anti-CpPGM1A recognized a weak and a strong band at positions at ~68 kDa and ~85 kDa that were larger than the calculated molecular weight of 63.2 kDa for this protein (Figure 5B). Western blot analysis for CpPGM1B was successful only when protein extracts in the sample buffer were treated at a lower temperature prior to SDS-PAGE fractionation (i.e., incubation at 37 °C for 10 min, rather than at 95 °C for 5 min—a condition commonly used in the western blot analysis of membrane proteins [22,23,24]), by which the antiserum recognized a very faint band at ~80 kDa and two major bands at ~180 kDa and at the entry of the separating gel (Figure 5B). The reasons for the abnormal gel migration of native CpPGM1A and CpPGM1B proteins were unknown, however the patterns were consistent and repeatable in multiple experiments. For CpPGM1A, the ~68 kDa band was close to the predicted molecular weight, although why it also migrated to the ~85 kDa position was unclear. A possible explanation may be that the protein was modified or covalently bound to a small molecule. For CpPGM1B, the faint band at ~80 kDa was close to the predicted molecular weight at ~75 kDa, and the band at ~180 kDa might be a dimer—a common form for native PGM1 proteins such as the parafusin-type PGM1 in *Paramecium tetraurelia* [25]. The band at the entry of separation gel was an indication of protein aggregation that was common for membrane proteins [23,24]. In fact, membrane-association of CpPGM1B was implied by IFA as described below. We have also ruled out the possibility of cross-reactivities of the two antibodies between CpPGM1A and CpPGM1B by western blot analysis using recombinant proteins (Figure S3).

In western blot analysis, fivefold more protein extracts were needed for CpPGM1B than for CpPGM1A to observe comparable signals (i.e., 2 × 10^6^ and 1 × 10^7^ oocysts per lane for CpPGM1A and CpPGM1B, respectively) (Figure 5B). This observation indicated that, in congruence with the qRT-PCR data on transcripts, CpPGM1A protein was also present at higher levels in the oocysts than those of CpPGM1B. The higher level of CpPGM1A than CpPGM1B in sporozoites was confirmed by dot blot analysis, in which the concentrations of the two primary antibodies were calibrated to the same titer to their peptide antigens by ELISA (Figure 5C, upper panel). However, dot blot analysis failed to detect the two proteins in samples prepared from intracellular parasites due to the low content of parasite proteins (vs. host cell proteins) (Figure 5C, lower panel). These observations also agreed with previously reported mass spectrum-based proteomic data, in which more peptide spectra were mapped to CpPGM1A than to CpPGM1B (Table S1) [26,27,28].

### 2.4. CpPGM1A and CpPGM1B Protein Were Differentially Distributed in the Sporozoites and Intracellular Parasites

**Figure 6:**
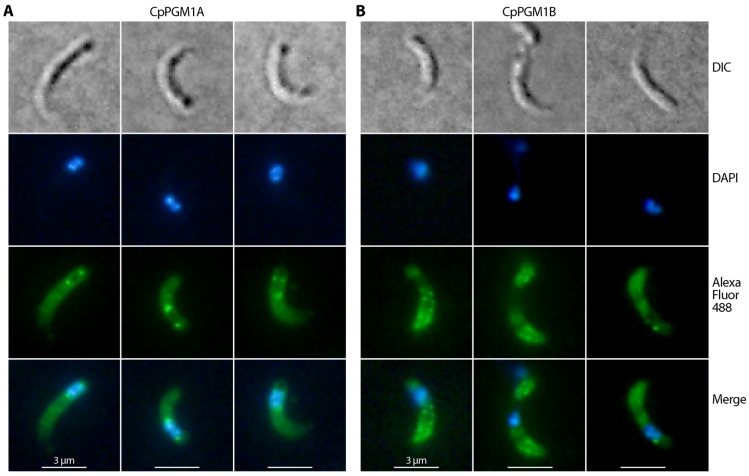
Immunofluorescence assay detection of CpPGM1A (**A**) and CpPGM1B (**B**) in the excysted sporozoites of *C. parvum* using primary antibodies as described in the text and a secondary antibody conjugated with Alexa Fluor 488 (green). Sporozoite morphology was observed by differential interference contrast microscopy (DIC). Nuclei were counterstained with 4, 6-diamidino-2-phenylindole (DAPI) (blue).

In free sporozoites, CpPGM1A was mainly localized to the cytosol, but also showed two concentrated spots adjacent to the frontier and posterior sides of the nuclei (Figure 6A). The cytosolic location was expected for CpPGM1A as a glycolytic enzyme, while the observation of two concentrated spots close to the nuclei was not expected to the same extent. Earlier ultrastructural analyses observed Golgi-like and putative mitochondrial relict structures [29,30,31]. Whether the two CpPGM1A-concentrated spots are correlated to these two structures remains to be resolved by immunogold electron microscopy and/or colocalization of CpPGM1A with appropriate markers in future studies. The significance of the enrichment of CpPGM1A at the two spots is also an intriguing question for future investigation. However, it is clear that the two spots are not correlated with amylopectin granules that are irregularly distributed in *C. parvum* sporozoites [32,33].

On the other hand, CpPGM1B was mainly distributed along the pellicles with some in granules in free sporozoites (Figure 6B), although we were unable to exclude its distribution in the cytosol as constrained by the resolution limitation of conventional IFA. The major fluorescent signals were defined within the sporozoite perimeters, suggesting a subpellicular distribution of CpPGM1B. Based on the prediction of the SP-containing CpPGM1B as a non-cytosolic protein and the pellicular structure, we speculated that CpPGM1B might be present in the inner membrane complex (IMC; also known as alveoli) that were flattened alveolar vesicles immediately under the plasma membranes [34]. However, the exact location of CpPGM1B and the biological significance remain to be resolved.

**Figure 7:**
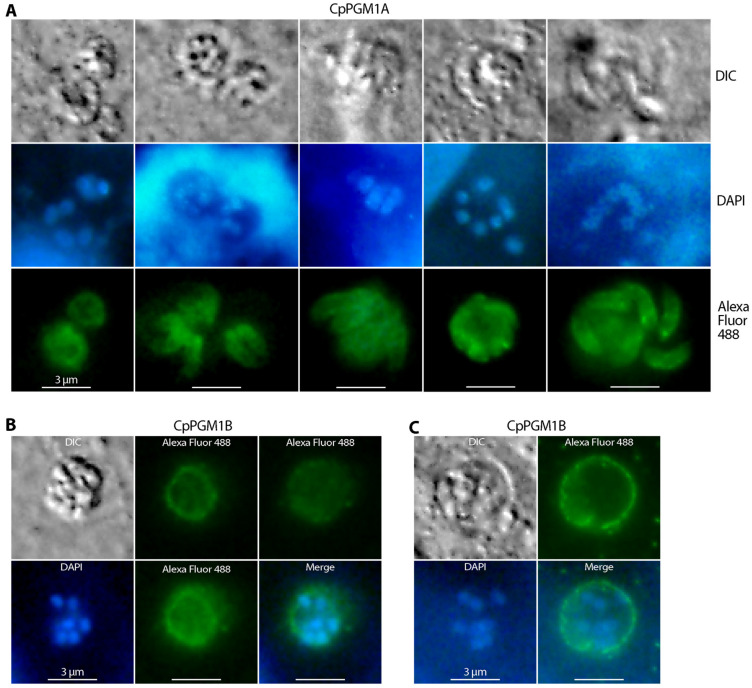
Immunofluorescence assay detection of CpPGM1A (**A**) and CpPGM1B (**B**,**C**) in the intracellular *C. parvum* using specified primary antibodies and a secondary antibody conjugated with Alexa Fluor 488 (green). Gross morphology was observed by differential interference contrast microscopy (DIC). Nuclei were counterstained with 4,6-diamidino-2-phenylindole (DAPI) (blue). In panel (**B**), Alexa Fluor 488 fluorescent images (green) were taken at three different focal points to indicate that major fluorescent signals were derived from the parasitophorous vacuole membrane (PVM) rather than from the merozoites within the PVM.

The two CpPGM1 isoforms were also differentially distributed in the intracellular stages of *C. parvum*. CpPGM1A became more concentrated to the membrane of developing meronts or developed merozoites, while relatively weaker cytosolic signals were present as well (Figure 7A). The relocation of cytosolic glycolytic enzymes was also observed in *T. gondii*, but in a different pattern. More specifically, upon egress of tachyzoites from host cells, some *Toxoplasma* glycolytic enzymes were relocated from the parasite’s cytoplasm to pellicles, probably to the cytoplasmic face of the IMC as well as to the space between the plasma membrane and IMC [35].

In the case of CpPGM1B, major fluorescent signals were observed in the PVM, although the presence or absence of signals in the merozoites enclosed by the PVM could not be fully resolved by the limitation of IFA (Figure 7B,C). For an N-terminal SP-containing protein that contained no transmembrane domains and was predictively co-translationally relocated into the ER lumen and secreted via secretory vesicles, CpPGM1B was expected to be located on the inner side of the PVM. The distribution on the PVM was previously observed for the lactate dehydrogenase of *C. parvum* (CpLDH), which was thought to facilitate the discharge of the organic end product lactate [20].

**Figure 8:**
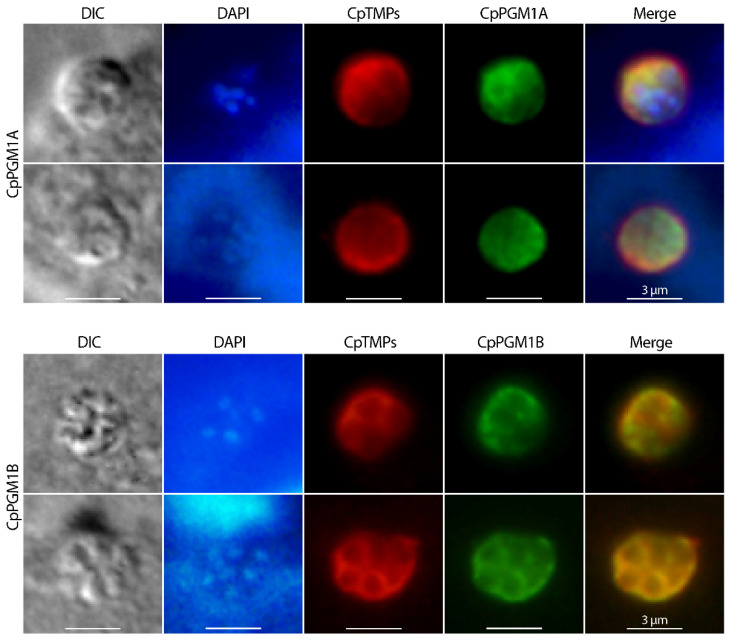
Immunofluorescence colocalization of CpPGM1A and CpPGM1B (green) in intracellular *C. parvum* with a mouse antiserum against *C. parvum* total membrane proteins (CpTMPs) (red) that stained both PVM and the residing parasites. CpPGM1A/CpPGM1B and CpTMPs were labeled with secondary antibodies conjugated with Alexa Fluor 488 and Alexa Fluor 596, respectively. The morphology was observed by differential interference contrast microscopy (DIC). Nuclei were counterstained with 4,6-diamidino-2-phenylindole (DAPI) (blue).

Similar patterns of IFA signals of CpPGM1A and CpPGM1B were observed in sporozoites and intracellular stages when specimens were fixed with cold methanol (−20 °C) without further permeabilization (Figure S4). The absence of CpPGM1A and the presence of CpPGM1B in PVM were also confirmed by colocalization of the two proteins with a mouse antiserum raised against *C. parvum* total membrane proteins (Figure 8).

This study observed differential expression of CpPGM1A at transcript and protein levels. There was an earlier enzyme motility study published in 1995 showing the presence of two PGM1 isoforms in 18 isolates of *C. parvum*, however only one isoform could be observed in the gel motility assay for individual isolates. Eight of the nine strains from humans (possibly also containing *C. hominis* based on current taxonomy) and those from cattle displayed higher and lower bands, respectively, suggesting the possibility that PGM1B (larger isoform) might be expressed at higher level in human isolates. However, we were unable to test this possibility using our antibodies due to the lack of *C. hominis* or *C. parvum* strains of human origin. Nonetheless, the differential locations of CpPGM1A and CpPGM1B indicated that these two PGM1 isoforms might play different roles in the parasite. The cytosolic or subpellicular location of the CpPGM1A protein within the parasites were indicative that CpPGM1A might play a house-keeping function. The subpellicular and PVM locations of CpPGM1B suggested that this protein played a less conventional role, however the exact biological role remains unclear.

In ciliates, parafusin is a member of the PGM superfamily with no phosphoglucomutase activity found to be associated with membrane and vesicle, or involved in the exocytic process [36,37,38]. In another apicomplexan, *T. gondii*, PGM1 (also termed as parafusin-related protein 1, PRP1; ToxoDB gene ID for ME49 strain: TGME49_285980) was first found to play a role in microneme exocytosis and cell invasion [39]. In a later study, PGM1 (PRP1) and PGM2 (TGME49_318580) were confirmed to be involved in Ca^2+^ -mediated microneme secretion, although the process was neither essential for lytic cycle, nor for acute virulence of *T. gondii* [40]. Based on these observations, it is intriguing to examine the potential role of the two *Cryptosporidium* PGMs, particularly the membrane associated CpPGM1B, in the exocytosis of the parasite in future studies.

## 3. Materials and Methods

### 3.1. The Parasite

A strain of *C. parvum* with subtype IIaA17G2R1 based on *gp60* gene was propagated in calves in-house. Oocysts in calf feces were purified by a sucrose gradient centrifugation protocol and stored at 4 °C as described in [28,41]. Prior to experiments, oocysts suspended in water were treated with 10% bleach on ice for 5 min to inactivate microbial contaminants, washed with water by centrifugation for 6 or more times, and examined for viability by an in vitro excystation protocol (i.e., incubation with 0.75% taurocholic acid in PBS at 37 °C for 45 to 60 min). Oocysts with >80% excystation rate were used in experiments.

### 3.2. Heterogenous Expression of Recombinant CpPGM1A and CpPGM1B Proteins

Genome DNA (gDNA) was isolated from *C. parvum* oocysts using a QIAamp DNA Mini Kit (Qiagen, Köln, Germany) and used for molecular cloning of *CpPGM1A* (locus cgd2_3260) and *CpPGM1B* (cgd2_3270) genes that contained no introns. For *CpPGM1A*, the entire open reading frame (ORF) was amplified from gDNA by PCR using a pair of primers CpPGM1A-F01 (5′-CGC GGA TCC ATG GAG CAA GTT CAA GT-3′) and CpPGM1A-R01 (5′-CCC AAG CTT TTA AGT TAT AAC AGT TGG CCT AT-3′). For *CpPGM1B*, two gene fragments were amplified by PCR: a long fragment containing the ORF with the exclusion of 63-nt N-terminal SP-encoding sequence at the 5′-end using primers CpPGM1B-F01L (5′-CGG ATC CGT GGG GAA TTT GAG ATA-3′) and CpPGM1B-R01L (5′-CAA GC TTT TAA GTA ATTA CTG TAG GCT TGC TTC TAC C-3′); and a short fragment with the exclusion of N-terminal SP and the following linker containing only the PGM1 subfamily domain using primers CpPGM1B-F01S (5′-CGG ATC CCT ATT AGA TAT GTT AGT TC-3′) and CpPGM1B-R01S (5′-CAA GCT TTT AAG TAA TTA CTG TAG G-3′). Note that underlined nucleotides in the primers were *Bam*HI or *Hin*dIII restriction linker sequences. PCR amplification used a 2 × M5 HiPer *Pfu* PCR MasterMix Kit (Mei5 Biotechnology Co., Beijing, China).

Amplified gene fragments were cloned into a pMD18-T vector using a T4 DNA Ligase (Sigma-Aldrich, St. Louis, MO, USA) and transferred into a DH5α strain of *Escherichia coli* (Kangti Life Technology Co., Shenzhen, China), followed by the isolation of plasmids from individual bacterial colonies using a M5 HiPer Multi-color Plasmid Miniprep Kit (Mei5 Biotechnology Co.). After sequencing, inserts with correct sequences were released by restriction enzyme digestion and subcloned into a pMAL-c5x vector (New England Biolabs, Ipswich, MA, USA) for expressing maltose-binding protein (MBP)-fusion proteins. The plasmids were sequenced again to ensure that insert sequences were correct and there were no ORF shifts. The final constructs were named pMAL-CpPGM1A, pMAL-CpPGM1B-L and pMAL-CpPGM1B-S, and their recombinant MBP-fusion proteins were named rCpPGM1A, rCpPGM1B-L and rCpPGM1B-S, respectively (Figure 1B).

The expression of recombinant proteins was carried out in BL21 (DE3) strain of *E. coli* cultured with 200 mL broth containing 0.2% glucose, and 100 μg/mL ampicillin at 37 °C until OD_600_ reached ~0.5–0.6. At this time point, the full name for IPTG (IPTG; 0.3 mM) was added to the culture to induce expression at 16 °C for 12 h. The purification of the recombinant proteins from the bacteria was performed using a NEBExpress MBP Fusion and Purification System kit according the manufacturer’s instruction (New England Biolabs, Ipswich, MA, USA). Purified proteins were subjected to 10% SDS-PAGE analysis for the determination of purity and quantified using a Bradford Protein Assay Kit (Beyotime Biotechnology Co., Beijing, China).

### 3.3. Determination of Enzyme Activity and Kinetics

A G6P-coupled reaction assay was used to detect the PGM enzymatic activity, in which the substrate G1P was converted by PGM to form G6P that was further oxidized to glucono-1,5-lactone-6P by G6P dehydrogenase (G6PDH) using the cofactor NAD^+^ as an electron acceptor. The formation of NADH was then detected spectrophotometrically by absorbance at 340 nm (Figure 1A). A typical reaction (final volume 200 μL in a microplate) was carried out in a 50 mM Tris-HCl buffer (pH8.5) containing 2 mM G1P, 0.2 mM NAD^+^, MgCl_2_ (1.0 mM), 2 units of G6PDH (Yuanye Bio-Technology Co., Shanghai, China), and one of the recombinant CpPGM proteins (200 ng). In negative controls, MBP-tag (200 ng) was used to replace the recombinant protein and for background subtraction. All reactions started with the addition of proteins and OD_340_ was measured every minute for 30 min in a multifunctional microplate reader (BioTek, Winooski, VT, USA). The optimal Mg^2+^ concentration for the reaction and the sensitivity of recombinant CpPGM1 enzymes on Li^+^ were determined in the same reaction with varied concentrations of Mg^2+^ (0.125 to 10 mM) or Li^+^ (15.6 to 1000 μM). For determining kinetic parameters towards substrate, G1P at varied concentrations was used in the reaction as specified, and the parameters including *K*_m_ and *V*_max_ were calculated using Prism (v8.0) (GraphPad Software, San Diego, CA, USA). All assays were independently performed at least three times, each with triplicated reactions.

### 3.4. Production of Polyclonal Antibodies against CpPGM1A and CpPGM1B

Both CpPGM1A and CpPGM1B are highly homologous with <10% mismatched amino acids, but there are two sites close to the C-terminus containing multiple mismatched amino acids for designing epitopes specific to individual proteins (Figure 1B and Figure S2). More specifically, two peptides unique to CpPGM1A and CpPGM1B (i.e., ^529^SDQTKVNSTSNEI^541^ and ^631^KNPQEFEKTTQQA^643^, respectively) with a Cys residue added to the N-terminus were synthesized by Qiangyao Biological Technology Co. (Shanghai, China) and conjugated to keyhole limpet hemocyanin (KLH) via maleimidobenzoyl-N-hydroxysuccinimide ester (MBS); the method is as described in [42]. To produce polyclonal antibodies, a rabbit was immunized by subcutaneous injection with a KLH-linked peptide (300 μg) emulsified with an equal volume of complete Freund’s adjuvant. There were three additional weekly injections of the KLH-linked peptide (150 μg) emulsified with incomplete Freund’s adjuvant. Pre-immune serum and antiserum were collected prior to the first injection and one week after the fourth injection. Due of the presence of a non-specific reaction, the antiserum to CpPGM1A was subjected to a membrane-based affinity purification using rCpPGM1A as the antigen as described in [42]. An antiserum or an affinity purified antibody were tested to rule out their potential cross-reactivity between CpPGM1A and CpPGM1B by western blot analysis using recombinant proteins. The animal use protocol was reviewed and approved by the Ethics Committee of Jilin University Institute of Zoonosis (AUP number IZ-2019-084; approval on 10 October 2019).

### 3.5. Western Blot and Dot Blot Analyses

The *C. parvum* oocysts were suspended in Pierce RIPA buffer (Thermo Fisher Scientific, Carlsbad, CA, USA) (10^8^ oocysts in 200 μL) containing 5 mM EDTA and 1× protease inhibitor cocktail (TransGen Biotechnology Co., Beijing, China). The suspension was subjected to six freeze/thaw cycles between liquid nitrogen and 37 °C water bath, incubated in ice overnight, and then at room temperature for 1 h. After centrifugation (500× *g* for 5 min), supernatant was collected, mixed with an equal volume of 2 × SDS-PAGE loading buffer, and heated at 95 °C for 5 min for CpPGM1A or 37 °C for 10 min for CpPGM1B, respectively. The lower temperature treatment for CpPGM1B was intended to reduce the aggregation of this membrane-associated protein.

After cooling down to room temperature, samples at specified concentrations were fractionated in 10% SDS-PAGE, transferred onto a nitrocellulose membrane (NC) membrane in a semi-dry transfer apparatus (Bio-Rad Laboratories, Hercules, CA, USA). Blots were blocked in a blocking buffer containing 20 mM Tris-HCl (pH 8.0), 0.05% Tween-20, 0.15 mM NaCl (TBST) and 5% skimmed milk for 1 h, followed by an incubation with affinity-purified anti-CpPGM1A antibody (1:30 dilution) or anti-CpPGM1B antiserum (1:400 or 1:800 dilution) in the blocking buffer. Their corresponding pre-immune antisera (subjected to the same affinity-purification procedure for CpPGM1A, or as original serum for CpPGM1B) were used as negative controls. Secondary antibody used a goat anti-rabbit IgG(H+L) conjugated with horseradish peroxidase (ImmunoWay Biotechnology Co., Plano, TX, USA) (1:5000 dilution in TBST buffer; 1 h). The blots were visualized using a Sensitive ECL Chemiluminescence Detection Kit according to the manufacturer’s protocol (Proteintech Group, Inc., Rosemont, IL, USA). There were three washes in TBST buffer at room temperature (5 min each) between steps. All procedures were conducted at room temperature or as specified.

For dot blot analysis, sporozoites were prepared by in vitro excystation as described above, and intracellular parasites were prepared by infecting HCT-8 cells in a 24-well cell culture plate for 12 h. Sporozoites and infected cells were lysed in RIPA buffer containing 5 mM EDTA and 1× protease inhibitor cocktail. After centrifugation (500× *g*), supernatants (10 μL/dot) were applied to nitrocellulose membranes (equivalent to 100,000 sporozoites per dot or 14 μg total proteins from infected cells per dot). After the membranes were airdried, they were subjected to blocking, incubated with affinity-purified anti-CpPGM1A antibody (1:5 dilution) or affinity-purified anti-CpPGM1B antibody (1:15 dilution), followed by procedures as described for western blot analysis. The concentrations of the two antibodies were calibrated to the same titer by ELISA using corresponding peptide antigens conjugated with BSA.

### 3.6. Immunofluorescence Microscopic Assay (IFA)

Free sporozoites of *C. parvum* were prepared by in vitro excystation as described above for oocyst viability assay, fixed in 4% paraformaldehyde for 30 min, washed with PBS by centrifugation, and applied to poly-L-lysin-treated microscopic slides. For parasite intracellular stages, host cells used a human ileocecal colorectal adenocarcinoma cell line HCT-8 (ATCC # CCL-244) that were cultured in 48-well plates containing round coverslips in RPMI 1640 medium with 10% fetal bovine serum at 37 °C under a 5% CO_2_ atmosphere until >70% confluence. Parasite oocysts were added into the plate (5 × 10^5^ oocysts per well) and allowed for excystation of sporozoites and invasion of host cells at 37 °C for 3 h. After a medium exchange to remove free parasites and oocyst walls, infected cell monolayers were allowed to grow for 24 h and then fixed with 4% paraformaldehyde for 30 min. Fixed sporozoites or monolayers were permeabilized with 0.1% Triton X-100 in PBS for 5 min, blocked with 3% BSA/PBS for 50 min, incubated with primary antibodies (i.e., purified anti-CpPGM1A antibody at 1:10 dilution or anti-CpPGM1B antiserum at 1:800 dilution) in 3% BSA/PBS at 4 °C overnight, labeled with goat anti-rabbit IgG conjugated with Alexa Fluor 488 (1:2000) (Invitrogen, Waltham, MA, USA) at 37 °C for 1 h, counterstained with 4′,6-diamidino-2-phenylindole (DAPI) at 1 μg/mL for 5 min, and mounted with an Antifade Mounting Medium (Beyotime Biotechnology). Specimens were examined under a BX53 research microscope (Olympus, Tokyo, Japan). For comparison, some sporozoite and intracellular parasite specimens were fixed with cold methanol (−20 °C) without further permeabilization, followed by the same IFA procedure as described.

### 3.7. Quantitative RT-PCR Detection of CpPGM1A and CpPGM1B Transcripts

Total RNA samples were isolated from *C. parvum* oocysts, excysted sporozoites and intracellular parasites cultured in HCT-8 cells at 3 h, 6 h, 12 h, 24 h, 48 h and 72 h post-infection (hpi) using an iScript qRT-PCR sample preparation reagent (lysis buffer) (Bio-Rad Laboratories). The relative transcripts of *CpPGM1A* and *CpPGM1B* genes were detected by a SYBR Green-based qRT-PCR using HiScript II One-Step qRT-PCR SYBR Green Kit (Vazyme Biotech Co., Nanjing, China) using the following primer pairs: 5′-TCC AGA GTT GTT TTC CGC CT-3′ and 5′-TGG CCT ATC TCT TCC TGT CG-3′ for *CpPGM1A* and 5′-CTC TCT CCG TTC AAG TGG GT-3′ and 5′-GTG ATT TAG AGC TTG TTG GGT TGT-3′ for *CpPGM1B*. The levels of *C. parvum* 18S rRNA were also detected using primers 5′-TAG AGA TTG GAG GTT GTT CCT-3′ and 5′-CTC CAC CAA CTA AGA ACG GCC-3′ for normalization as described [43,44]. As a quality control, the transcript of a previously characterized lactate dehydrogenase (*CpLDH*) gene was also detected using primers as described [20,21]. Each reaction in a 20 µL final volume contained 0.2 µM of each primer, 1.0 µL One Step SYBR enzyme mix, 10 µL SYBR Green mix, and 0.4 µL ROX reference dye 1 (50×), 0.2 ng of total RNA isolated from oocysts/sporozoites or 15 ng total RNA isolated from intracellular parasites (Vazyme Biotech). Thermal cycling started with 50 °C for 3 min to synthesize cDNA, followed by incubation at 95 °C for 30 s for inactivate the reverse transcriptase and 40 cycles at 95 °C for 10 s and 60 °C for 30 s to produce amplicons. At least two technical replicated qRT-PCR reactions were performed for each sample. Relative transcript levels were calculated using an empirical 2^(−∆∆C_T_) formula as described [45].

### 3.8. Phylogenetic Reconstruction

To gain inside knowledge of the evolutionary history of CpPGM1A and CpPGM1B, we performed a phylogenetic reconstruction of PGM proteins from apicomplexans and other taxonomic groups. A total of 606 PGM protein sequences from major taxonomic groups were retrieved from the GenBank protein or EuPathDB databases using CpPGM1A sequence as a query. Sequences were aligned using MUSCLE program (v3.8.31; http://www.drive5.com/muscle) (accessed on 23 December 2021) and redundant or incomplete sequences were removed, from which a final of 94 representative sequences from major taxonomic groups were retained for subsequent phylogenetic reconstruction, including those from apicomplexans (*n* = 20), chromerida (*n* = 2), ciliates (*n* = 4), plants (*n* = 32), animals (*n* = 20), and prokaryotes (*n* = 18). A Bayesian inference-based phylogenetic reconstruction was conducted using the MrBayes program (v3.2.6; http://nbisweden.github.io/MrBayes) (accessed on 23 December 2021). Amino acid substitutions used a *WAG* model with the consideration of the fraction of invariable sites and four discrete rates of gamma distribution (*WAG* + *F*_inv_ + *Γ*_[rate = 4]_). Two independent searches (each with four chains) were run for one million generations, and trees were sampled in every thousand generations of runs. A consensus tree with posterior probabilities summarized the bottom 75% of the sampled trees. FigTree (v1.4.4; http://tree.bio.ed.ac.uk/software/figtree) (accessed on 23 December 2021) and Adobe Illustrator (v25 or higher; https://www.adobe.com) (accessed on 23 December 2021) were used to display and annotate the consensus tree.

## 4. Conclusions

*Cryptosporidium* parasites possess two tandemly duplicated *PGM1* genes, in which *PGM1A* encodes a conventional PGM1 protein (PGM1 domain only), while *PGM1B* encodes a PGM1 containing an N-terminal SP and a linker sequence upstream to the PGM1 domain (Figure 1B). This feature differs from other major groups of apicomplexans that contain none or only a single *PGM1* gene. In the zoonotic species *C. parvum*, both CpPGM1A and CpPGM1B are enzymatically active and able to catalyze the interconversion between G1P and G6P. The transcript and protein product of the *CpPGM1A* gene are present at much higher levels than those of *CpPGM1B* during the parasite life cycle stages. Both the CpPGM1A and the CpPGM1B proteins are differentially located in the parasite sporozoites and intracellular meronts, indicating that these two isoforms play differential biological roles in the parasite.

## Supplementary Materials

The following are available online at https://www.mdpi.com/article/10.3390/pathogens11010021/s1, Figure S1: Sequence comparison between CpPGM1A and CpPGM1B proteins and annotation of active site residues. Figure S2: Comparison between CpPGM1A and CpPGM1B at nucleotide and protein levels. CpPGM1A and CpPGM1B genes are tandemly duplicated. The two orthologs are highly conserved at both protein and nucleotide levels. Figure S3: Test of cross-reactivity of the two antibodies between CpPGM1A and CpPMG1B by western blot analysis using recombinant proteins. Figure S4: IFA labeling of CpPGM1A and CpPGM1B in sporozoites and intracellular parasites. Samples were fixed with cold methanol. Table S1: Counts of CpPGM1A and CpPGM1B spectra in mass spectrum-based proteomics.
