# Supplementary material for: Implication of Potential Differential Roles of the Two Phosphoglucomutase Isoforms in the Protozoan Parasite Cryptosporidium parvum
Source: Pathogens. 2021 Dec 24;11(1):21. doi: 10.3390/pathogens11010021 (PMC8781159; doi:10.3390/pathogens11010021)
Supplement: Supplementary file 1 [file pathogens-11-00021-s001.zip › figure S1.pdf]

Figure S1. Sequence comparison between CpPGMIA and CpPGM1B proteins and annotation of active site residues

```
CpPGM1B  MMKILNILFSLVLVSQYVNANESVGNLRYLQKINLIEPTDGQAKVVTSQD  50

CpPGM1B  VQINQGRALIKKKVSVVTRNVIETETTETYYYSDKGLDAWSPNENDNLLD  100

CpPGM1A    MEQVQVTPYLDQKPGTSGLRKKTRVFMEGTYLANFIESYFQSFPPENF  48
CpPGM1B  MLVQEVQISPYLDQKPGTSGLRKKTRVFMEGTYLANFIESYFQSFPPENF  150

CpPGM1A  EGATLLVAGDGRFFLPEAIQIISEIAAAHKVKRIWTGVNGLCSTPAGSAI  98
CpPGM1B  EGATLLVAGDGRFFLPEAIQIISEIAAAHKVKRIWTGVNGLCSTPAGSAI  200

CpPGM1A  IREREGGIAVGGILLTASHNPGGIDEDFGVKFNGKNGGPAQDSVTNTIFE  148
CpPGM1B  IREREGGIAVGGILLTASHNPGGIDEDFGVKFNEKNGGPAQDSVTNAIFE  250

CpPGM1A  ITKKLTSYKKISLPKIDLSKIGVQELIPNQFTVEVIDTSEDWLKLMKKIF  198
CpPGM1B  ITKKLTSYKKISLPKIDLSKIGVQELIPNQFTVEVIDTSEDWLKLMKKIF  300

CpPGM1A  DFQKIQNLLNRKDFKMAFDSMHGVAGPYARKLFVDEFGLPESSLLHLESK  248
CpPGM1B  DFQKIQNLLNRKDFKMVFDSMHGVAGPYARKLFIDEFGLPESSLLHLESK  350

CpPGM1A  PDFGGLHPDPNLTYAKDLVELMKVKSPEKVDKSTPDFGAAGDGDCDRNMI  298
CpPGM1B  PDFGGLHPDPNLTYAKDLVELMKVKSPEKVDKNTPDFGAAGDGDCDRNMI  400

CpPGM1A  LGKGFFVTPSDSVAIIASYAKEAIPYFSKGLAGVSRSMPTSTSLNNVAEK  348
CpPGM1B  LGKGFFVTPSDSVAIIASYAKEAIPYFSKGLAGVSRSMPTSTSLNNVAEK  450

CpPGM1A  LGIPCYEVPTGWKYFGNLMDAKMIDICGEESFGTGSDHIREKDGLWAVLA  398
CpPGM1B  LGIPCYEVPTGWKYFGNLMDAKMIDICGEESFGTGSDHIREKDGLWAVLA  500

CpPGM1A  WLSILAHKNPDPTKPLVSVEDITREFWKTYGRNYYTRFDYESIETEKADQ  448
CpPGM1B  WLSILAYKNPDPTKSLVSVEDITREFWKTYGRNYYTRFDYESVETEKADQ  550

CpPGM1A  FFKHLNSLMEDHQKLREIIKPYGIDIKLTDNFTYNDPVDKSVAKNQGLRF  498
CpPGM1B  FFKHLNSLMEDSQKLRNSLRSSGLKVKFMDNFTYNDPVDGSVTKNQGVRI  600

CpPGM1A  IFQDNSRVVFRLSGTGSVGATIRIYIEKTVSDQTKVNSTSNEILGDLIEI  548
CpPGM1B  IFTDGSRIIFRISGTGSVGATIRVYMEKTVKNPQEFEKTTQQALNHLIEI  650

CpPGM1A  VEKKIKLKESTGRDRPTVIT  568
CpPGM1B  VEKKIKLKEITGRSKPTVIT  670
```

Note: X (underlined) = Conserved active site residue based on structure 1KFI_A (*Paramecium tetraurelia* parafusin)

X (boxed) = Conserved metal (Mg^{2+}) binding site residues based on structure 3PMG_A (*Oryctolagus cuniculus* Phosphoglucomutase)

X (yellow) = Conserved substrate binding site residue based on the structures of *Candida albicans* AGM1 (N-Acetylglucosamine-phosphate mutase) and *Pseudomonas aeruginosa* PMM/PGM bound to their substrates.

X (green) = Mismatched residues between CpPGM1A and CpPGM1B
