# Supplementary material for: Implication of Potential Differential Roles of the Two Phosphoglucomutase Isoforms in the Protozoan Parasite Cryptosporidium parvum
Source: Pathogens. 2021 Dec 24;11(1):21. doi: 10.3390/pathogens11010021 (PMC8781159; doi:10.3390/pathogens11010021)

Figure S2

Comparison between CpPGM1A and CpPGM1B at nucleotide and protein levels, showing that *CpPGM1A* and *CpPGM1B* genes are tandemly duplicated. They are highly conserved at both protein and nucleotide levels

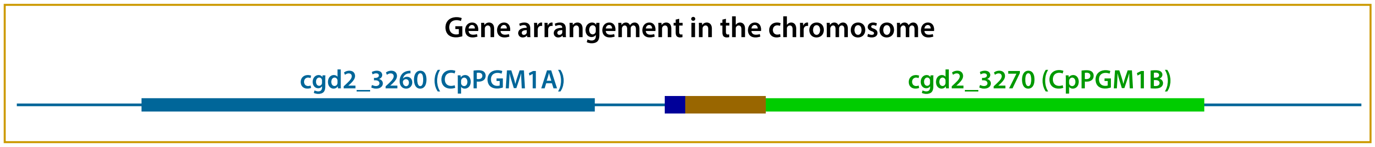

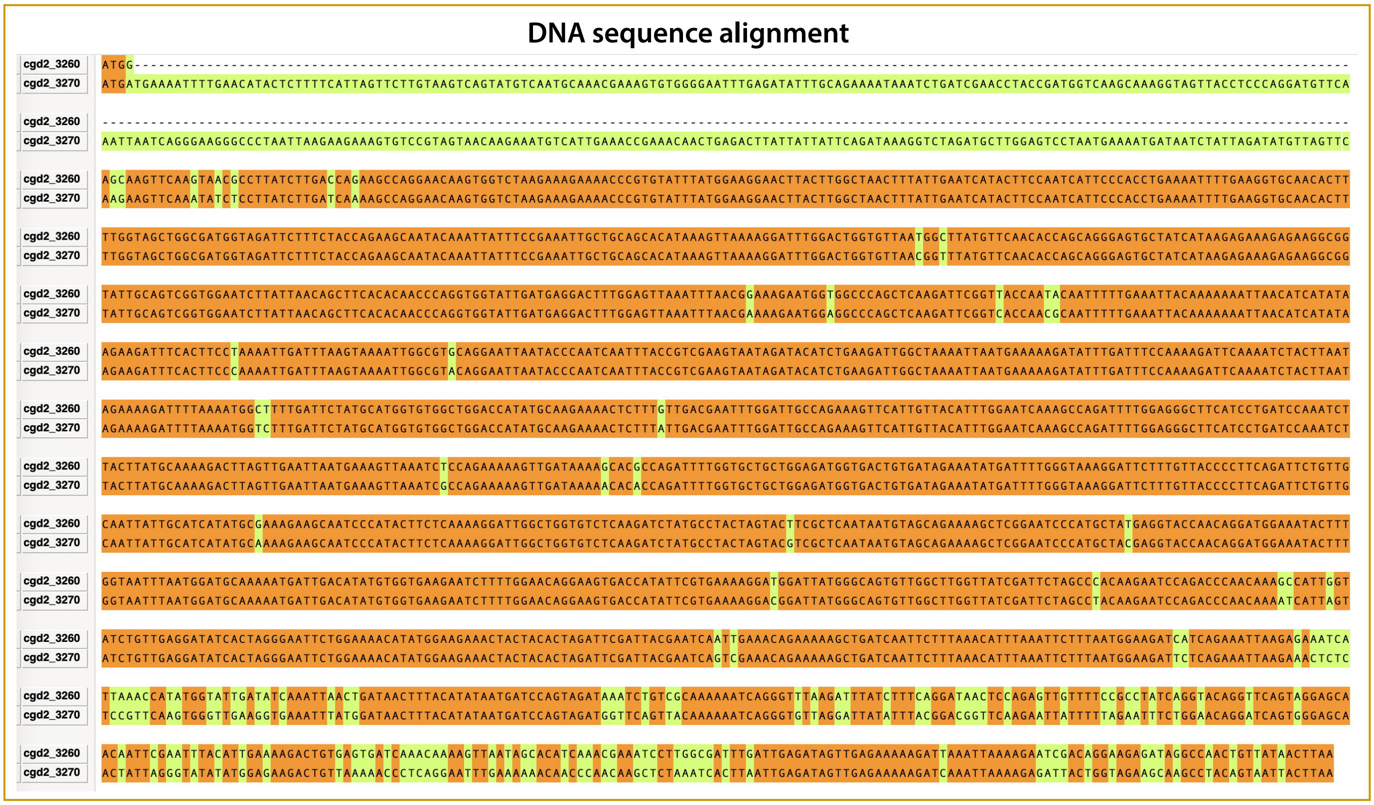

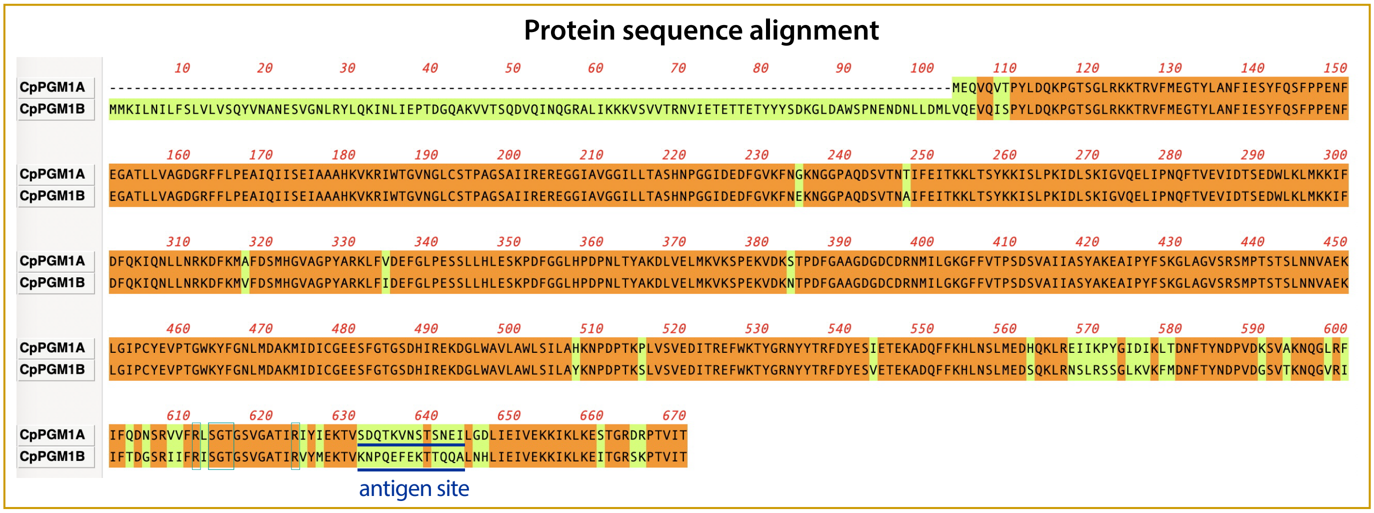

Supplement: Supplementary file 1 [file pathogens-11-00021-s001.zip › figure S2.pdf]
