# Supplementary material for: Implication of Potential Differential Roles of the Two Phosphoglucomutase Isoforms in the Protozoan Parasite Cryptosporidium parvum
Source: Pathogens. 2021 Dec 24;11(1):21. doi: 10.3390/pathogens11010021 (PMC8781159; doi:10.3390/pathogens11010021)

Figure S3.

Test of cross-reactivity of the two antibodies between CpPGM1A and CpPMG1B by western blot analysis using recombinant proteins.

A) SDS-PAGE of CpPGM1A and CpPGM1B-S recombinant proteins.

B) Western blot analysis of recombinant CpPGM1A for reactivity with antibodies against CpPGM1A or CpPGM1B.

C) Western blot analysis of recombinant CpPGM1B for reactivity with antibodies against CpPGM1B or CpPGM1A.

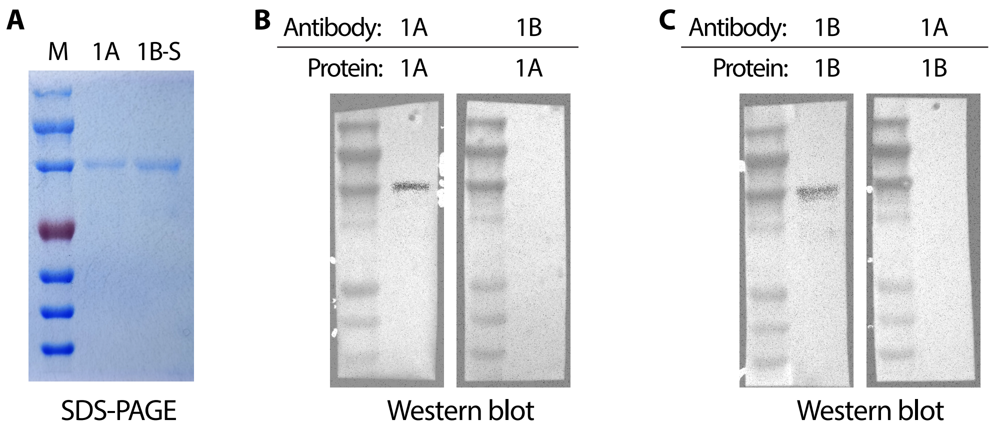

Supplement: Supplementary file 1 [file pathogens-11-00021-s001.zip › figure S3.pdf]
