# Supplementary material for: Implication of Potential Differential Roles of the Two Phosphoglucomutase Isoforms in the Protozoan Parasite Cryptosporidium parvum
Source: Pathogens. 2021 Dec 24;11(1):21. doi: 10.3390/pathogens11010021 (PMC8781159; doi:10.3390/pathogens11010021)

Figure S4.

Immunofluorescence assay (IFA) labeling of CpPGM1A and CpPGM1B in sporozoites (**A**) and intracellular parasites (**B**). Samples were fixed with cold methanol. **DIC**, differential phase contrast microscopy; **DPAI**, 4,6-diamidino-2-phenylindole (for counter-staining nuclei); **Alexa Fluo 488**, fluorophore used for IFA-labeling of CpPGM1A or CpPGM1B protein

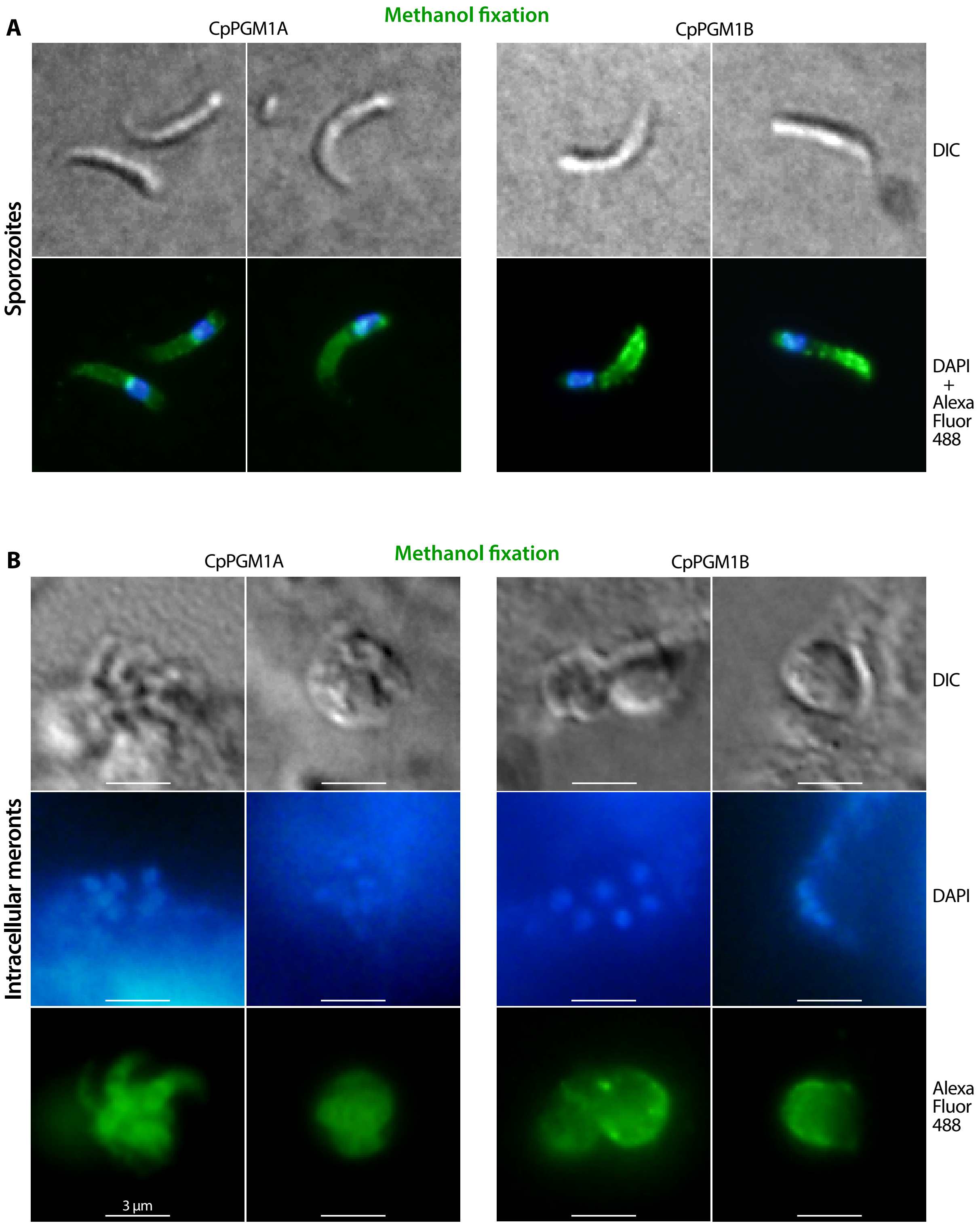

Supplement: Supplementary file 1 [file pathogens-11-00021-s001.zip › figure S4.pdf]
