# Supplementary material for: Implication of Potential Differential Roles of the Two Phosphoglucomutase Isoforms in the Protozoan Parasite Cryptosporidium parvum
Source: Pathogens. 2021 Dec 24;11(1):21. doi: 10.3390/pathogens11010021 (PMC8781159; doi:10.3390/pathogens11010021)
Supplement: Supplementary file 1 [file pathogens-11-00021-s001.zip › Table S1.pdf]

Table S1. Counts of CpPGM1A and CpPGM1B in mass spectrum-based proteomics*

Experiment	Sample	CpPGM1A		CpPGM1B	
		Sequences	Spectra	Sequences	Spectra
Enriched cytoskeletal & membrane fractions	1D Gel 16May2006	6	20	3	8
Enriched cytoskeletal & membrane fractions	1D Gel 24Jun2006	2	3	1	2
Oocyst Wall proteome (IowaII)	Intact Oocysts	16	33	0	0
Oocyst Wall proteome (IowaII)	Oocyst walls	9	15	0	0
Oocyst Wall proteome (IowaII)	Sporozoites	13	30	0	0
Proteome during sporozoite excystation	Soluble excysted/non-excysted LC-MS/MS	9	50	0	0
Sporozoite proteome (IowaII)	1D Gel LC-MS/MS	22	57	3	3
Sporozoite proteome (IowaII)	2D Gel LC-MS/MS	5	9	0	0
Sporozoite proteome (IowaII)	MudPit insoluble fractions	7	7	8	11
Sporozoite proteome (IowaII)	MudPit soluble fractions	21	32	0	0
**Total counts**		**110**	**256**	**15**	**24**

*Data were extracted from CryptoDB (https://www.cryptodb.org)
